# Appropriateness of antibiotic use in patients with and without altered mental status diagnosed with a urinary tract infection

**DOI:** 10.1017/ash.2022.346

**Published:** 2022-12-13

**Authors:** Connor M. Anderson, Jeremy D. VanHoose, Donna R. Burgess, David S. Burgess, Aric Schadler, J. Zachary Porterfield, Katie L. Wallace

**Affiliations:** 1 University of Kentucky HealthCare, Lexington, Kentucky; 2 University of Kentucky College of Pharmacy, Lexington, Kentucky; 3 University of Kentucky College of Medicine, Lexington, Kentucky

## Abstract

**Objective:**

The objective of this study was to determine antibiotic appropriateness based on Loeb minimum criteria (LMC) in patients with and without altered mental status (AMS).

**Design:**

Retrospective, quasi-experimental study assessing pooled data from 3 periods pertaining to the implementation of a UTI management guideline.

**Setting:**

Academic medical center in Lexington, Kentucky.

**Patients:**

Adult patients aged ≥18 years with a collected urinalysis receiving antimicrobial therapy for a UTI indication.

**Methods:**

Appropriateness of UTI management was assessed in patients prior to an institutional UTI guideline, after guideline introduction and education, and after implementation of a prospective audit-and-feedback stewardship intervention from September to November 2017–2019. Patient data were pooled and compared between patients noted to have AMS versus those with classic UTI symptoms. Loeb minimum criteria were used to determine whether UTI diagnosis and treatment was warranted.

**Results:**

In total, 600 patients were included in the study. AMS was one of the most common indications for testing across the 3 periods (19%–30.5%). Among those with AMS, 25 patients (16.7%) met LMC, significantly less than the 151 points (33.6%) without AMS (*P* < .001).

**Conclusions:**

Patients with AMS are prescribed antibiotic therapy without symptoms indicative of UTI at a higher rate than those without AMS, according to LMC. Further antimicrobial stewardship efforts should focus on prescriber education and development of clearly defined criteria for patients with and without AMS.

Treatment of asymptomatic bacteriuria continues to lead to unnecessary antibiotic use. In a 2019 study by Petty et al,^1^ older age, dementia, and acutely altered mental status (AMS) were associated with treatment of asymptomatic bacteriuria. Treatment of asymptomatic bacteriuria was associated with longer duration of hospitalization after urine testing with no significant differences in outcomes including 30-day postdischarge mortality, readmission, and emergency department (ED) visits. Furthermore, AMS lacks definitive evidence to be linked with urinary tract infection (UTI).^2^

The Society for Healthcare Epidemiology of America and Infectious Diseases Society of America have endorsed expert guidance regarding nonlocalizing signs and symptoms as indicators of infection in nursing home residents.^3^ This guidance emphasizes that better identification of true delirium to assist in UTI workups in this patient population is needed. Furthermore, there are generally accepted criteria for defining symptomatic UTI for long-term care facility residents without an indwelling catheter that use symptom-based evaluation for starting antimicrobial therapy including acute dysuria, fever, various urinary symptoms, hematuria, and costovertebral angle tenderness.^4^ These are referred to as the Loeb minimum criteria (LMC), shown in Table 1. Delirium is included in these criteria if an indwelling catheter is also present, making this the first published criteria set incorporating a form of AMS. In this study, we sought to determine antibiotic appropriateness based on LMC in patients with and without AMS using data from a UTI antibiotic stewardship initiative implemented at our institution.

**Table 1.** Loeb Minimum Criteria^4^

No Indwelling Catheter Present	Indwelling Catheter Present^a^
Either of the following constitute an indication for antibiotic treatment: 1. Acute dysuria alone OR Fever (>37.9°C [100°F] or 1.5°C [2.4°F] increase above baseline temperature)^b^ plus at least 1 of the following: • New or worsening urgency • New or worsening frequency • New or worsening suprapubic pain • Gross hematuria • Costovertebral angle tenderness • Urinary incontinence	Presence of one or more of the following constitutes an indication for antibiotic treatment: • Fever (>37.9°C [100°F] or 1.5°C [2.4°F] increase above baseline temperature)^¶^ • New costovertebral angle tenderness • Rigors (shaking or chills) with or without identified cause • New onset delirium

a Indwelling Foley or suprapubic catheter.

b 1.5°C [2.4°F] increase above baseline temperature not used for the purposes of this study.

## Methods

### Study design and patients

A UTI stewardship initiative was implemented at University of Kentucky HealthCare (UKHC), which consists of 2 main hospitals, Albert B. Chandler Medical Center and Good Samaritan Hospital, both located in Lexington, Kentucky. Albert B. Chandler Medical Center is a large, academic, tertiary-care facility with 1,040 licensed beds, and Good Samaritan Hospital is an acute-care facility with 180 licensed beds. This stewardship initiative was implemented due to retrospective data demonstrating low rates of guideline-adherent management in patients evaluated between September and November 2017 (preguideline group). An institutional UTI guideline was implemented in July 2018 and education was provided. Patients evaluated between September and November 2018 were referred to as the postguideline group. A prospective audit-and-feedback initiative was then implemented in June 2019, and patients evaluated between September and November 2019 were referred to as the prospective audit-and-feedback group. In total, 200 patients in each group were evaluated for guideline adherence and the indication for obtaining a urinalysis was obtained in all 600 patients. Patients meeting inclusion criteria were randomly selected using the Excel (Microsoft, Redmond, WA) randomization function, from which the first 200 randomized patients were selected within each period. AMS was the primary indication for UTI workup in the majority of the 3 groups (19%–30.5%). Patients with AMS were then directly compared to patients without AMS for the purpose of this study. Patients >18 years of age with a urinalysis and actively receiving antimicrobial therapy for a UTI indication were included. We applied the following exclusion criteria: pregnancy, undergoing a urologic procedure, leaving against medical advice, or death during UTI treatment, transfer from an outside hospital on therapy for a UTI indication, on suppressive antimicrobial therapy for UTI prevention, or receiving antimicrobial therapy for a concomitant infection.

### Outcomes measures and definitions

The primary outcome was to compare the percent of patients qualifying for LMC between patients with and without AMS. Qualifying symptoms were screened and were accounted for within the extracted data set. Manual patient electronic health record review was conducted when deemed necessary by the research committee to confirm presence of symptoms. Delirium was evaluated using criteria listed in the *Diagnostic and Statistical Manual of Mental Disorders* (DSM-5).^5^ Delirium was considered present when at least 3 of the 5 DSM-5 diagnostic criteria for delirium were noted through diagnosis code and keyword search in the physician’s progress notes.

Secondary outcomes included total days of therapy, 90-day all-cause mortality from patient admission, length of stay, ICU admission, ICU length of stay, qualifying symptoms for UTI based on LMC, other complicating factors, true pyuria (defined by urinalysis with a white blood cell count >10 per high-power field), and antibiotic ranking. Antibiotic rankings were assigned using the Duke Antimicrobial Stewardship Outreach Network (DASON) antibiotic ranking system (Table 2).^6^

**Table 2.** DASON Antibiotic Classification

Included Antimicrobial Agents		
Broad Spectrum^a^	Extended Spectrum, Including MDRO and *Pseudomonas*	Protected
Amoxicillin/clavulanate Ampicillin/sulbactam	Aminoglycosides	Anti-pseudomonal carbapenems
Azithromycin	Aztreonam	Ceftaroline
Ceftriaxone	Cefepime	Ceftazidime-avibactam Ceftolozane-tazobactam Colistin
Clarithromycin	Ceftazidime	Daptomycin
Clindamycin	Ertapenem	Linezolid
	Fluoroquinolones	Tedizolid
	Anti-pseudomonal penicillins	Tigecycline
	Vancomycin	

a Cefdinir and fosfomycin not included. For the purposes of this study, each was classified as broad spectrum.

### Data collection

Data were extracted from 3 separate sources. Patient data were obtained through the UKHC electronic health record, Sunrise Clinical Manager (Integrated Health Systems, Singapore). Demographic data were obtained from the Enterprise Data Warehouse with assistance of the Center for Clinical and Translational Science (CCTS), and patients on an antibiotic with a UTI indication were identified using a Tableau dashboard. Study data were collected and managed using Research Electronic Data Capture (REDCap) electronic data capture tools hosted at University of Kentucky Healthcare. REDCap is a secure, web-based software platform designed to support data capture for research studies. It provides (1) an intuitive interface for validated data capture; (2) audit trails for tracking data manipulation and export procedures; (3) automated export procedures for seamless data downloads to common statistical packages; and (4) procedures for data integration and interoperability with external sources.

### Statistical analysis

Bivariate analysis was used to compare the AMS and No AMS groups. Based on the structure of the data, a Mann-Whitney *U* test was used to analyze continuous variables. A Pearson χ^2^ test or Fisher exact test was used for nominal variables as appropriate to evaluate significant baseline differences. An α level of <0.05 was considered statistically significant. All statistical analyses were performed using IBM SPSS Statistics version 28 software (IBM, Armonk, NY).

## Results

### Baseline characteristics

In total, 600 patients were included in the study; 450 presented without AMS and 150 presented with AMS. Patient age ranged from 18 to 104 years, with a mean of 61.4 years, and most were female (70.3%). When compared by period, patients were significantly older in the prospective audit-and-feedback group (64.4 years vs 60.6 and 59.2 years; *P* = .014). There were no significant differences in patient sex or body weight among the 3 groups. The incidences of diabetes mellitus with end organ damage and mild liver disease were higher in the postguideline group. All other Charlson comorbidity index conditions were not statistically different between groups. Compared to those without AMS, patients with AMS were older (71.5 years vs 62.0 years; *P* < .001), had a larger proportion of females to males (76.7% vs 68.2%; *P* = .050) and had 1 more comorbidity on average (5.0 vs 4.0; *P* = .001). The 3 comorbid conditions identified to have occurred more frequently in the AMS population were cardiovascular disease, dementia, and diabetes without chronic complication. No statistically significant differences were observed between those with or without AMS based on the presence of an indwelling catheter and the remaining comorbid conditions listed in Table 3 in accordance with the Charlson comorbidity index.

**Table 3.** Baseline Characteristics

Characteristic	No AMS (n = 450), No. (%)^a^	AMS (n = 150), No. (%)^a^	*P* Value
Age, y^b^	62.0 (45.0–72.3)	71.5 (61.6–81.0)	<.001
Sex, female	307 (68.2)	115 (76.7)	.050
Indwelling catheter	135.0 (30.0)	43.0 (28.6)	.757
**Charlson-comorbidity index** ^**b**^	4.0 (2.0–7.0)	5.0 (4.0–7.0)	.001
Myocardial infarction^c^	63 (14.0)	18 (12.2)	.565
Heart failure^c^	74 (16.5)	26 (17.6)	.759
Cardiovascular disease^c^	74 (16.5)	48 (32.4)	<.001
Peripheral vascular disease^c^	46 (10.2)	14 (9.5)	.783
Dementia^c^	34 (7.6)	34 (23.0)	<.001
Chronic obstructive pulmonary disease^c^	106 (23.6)	35 (23.6)	.992
Peptic ulcer disease^c^	14 (3.1)	7 (4.7)	.356
Mild liver^c^	77 (17.1)	23 (15.5)	.649
Moderate or severe liver disease^c^	26 (5.8)	10 (6.8)	.669
Diabetes without chronic complication^c^	125 (27.8)	56 (37.8)	.022
Diabetes with chronic complication^c^	91 (20.3)	30 (20.3)	.999
Hemiplegia or paraplegia^c^	57 (12.7)	16 (10.8)	.544
Renal Disease^c^	102 (22.7)	42 (28.4)	.163
Cancer^c^	34 (7.6)	10 (6.8)	.742
Metastatic solid tumor^c^	23 (5.1)	6 (4.1)	.600
AIDS/HIV^b^	1 (0.2)	0 (0)	…

Note. HPF, high-power field.

a Units unless otherwise indicated.

b Median [interquartile range].

c 449 in no-AMS group and 148 in AMS group due to missing data….

### Primary outcome

Primary and secondary outcomes are shown in Table 4. Of the patients with AMS, 25 (16.7%) met the LMC, which was significantly fewer than the 151 (33.6%) without AMS who met the LMC (*P* < .001). In total, 39 patients (6.5%) met the LMC in the preguideline group; 68 patients (11.3%) in the postguideline group and 69 (11.5%) in the prospective audit-and-feedback group met the LMC. The criteria qualifying each patient for LMC are listed in Table 5. The most frequent criteria met in either category was acute dysuria followed by the presence of an indwelling catheter and fever. Delirium accounted for only 4 of 25 patients who met the LMC in the AMS group.

**Table 4.** Primary and Secondary Outcomes

Outcome	No AMS Group (n = 450), No. (%)^a^	AMS Group (n = 150), No. (%)^a^	*P* Value
**Primary outcome**			
Met Loeb criteria	151 (33.6)	25 (16.7)	<.001
**Secondary outcomes**			
Median total days of therapy	5.0 (3.0–7.0)	5.0 (3.0–7.0)	.313
90-day mortality	22 (4.9)	14 (9.3)	.047
Median length of stay, d	6.0 (4.0–11.3)	5.0 (3.0–10.0)	.347
ICU admission	32.0 (7.1)	8.0 (5.4)	.460
ICU length of stay, d	6.5 (3.0–13.3)	7.0 (3.0–13.0)	.776
**UTI symptoms**			
Dysuria	117 (26.0)	15 (10)	<.001
Increased urinary frequency	45 (10.0)	7 (4.7)	.044
Increased urinary urgency	24 (5.3)	3 (2.0)	.111
Unexplained suprapubic or flank pain	90 (20.0)	11 (7.3)	<.001
Fever >38°C	74 (16.4)	20 (13.3)	.364
**Complicating factors**			
N/A	223 (49.6)	74 (49.3)	.962
Urostomy	6 (1.3)	3 (2.0)	.698
Nephrostomy tubes	6 (1.3)	1 (0.7)	.687
Stent	12 (2.7)	4 (2.7)	1.000
Kidney transplant recipient	17 (3.8)	4 (2.7)	.617
History of recurrent UTI	36 (8.0)	25 (16.7)	.002
Other	53 (11.8)	17 (11.3)	.883
UA with WBC >10/HPF	344 (76.4)	125 (83.3)	.077
Urine culture obtained	345 (76.7)	129 (86.0)	.015
**Empiric antibiotic rank per DASON**			.582
Narrow spectrum	5 (1.1)	2 (1.3)	
Broad spectrum	75 (16.7)	19 (12.7)	
Extended spectrum, MDRO and PSA	258 (57.3)	97 (64.7)	
Protected	13 (2.9)	3 (2.0)	

Note. AMS, altered mental status; ICU, intensive care unit; UTI, urinary tract infection; N/A, not available; UA, urinalysis; WBC, white blood cell; HPF, high-power field; MDRO, multidrug-resistant organism; PSA, *Pseudomonas aeruginosa*.

a Units unless otherwise indicated.

**Table 5.** Patients Qualifying for Loeb Minimum Criteria by Criteria Met

Loeb Criteria Met	No AMS (n = 151), No. (%)	AMS (n = 25), No. (%)
Acute dysuria	92 (60.9)	11 (44.0)
Indwelling catheter & fever	26 (17.2)	6 (24.0)
Indwelling catheter & unexplained suprapubic/flank pain	14 (9.3)	0 (0)
Indwelling catheter & likely delirium	0 (0)	4 (16.0)
Fever & increased urinary urgency	2 (1.32)	2 (8.0)
Fever & incontinence	0 (0)	1 (4.0)
Fever & gross hematuria	1 (0.6)	1 (4.0)
Fever & unexplained suprapubic/flank pain	14 (9.3)	0 (0)
Fever & increased urinary urgency	2 (1.3)	0 (0)

### Secondary outcomes

We did not detect significant differences between patients with AMS and those without AMS for total days of antibiotic therapy, length of stay, or ICU admission or length of stay. The 90-day mortality rate was significantly greater in the AMS group (9.3% vs 22.0%; *P* = .047). Most antibiotics used in both groups were classified as extended-spectrum, multidrug-resistant organism (MDRO) antibiotics and antipseudomonal antibiotics: 64.7% in those with AMS and 57.3% in those without AMS. Dysuria was the most common qualifying symptom for patients without AMS (26.0%), followed by unexplained suprapubic or flank pain (20.0%). In the AMS group, the most common qualifying symptoms included fever (13.3%), followed by dysuria (10.0%). The differences in the presence of dysuria and unexplained suprapubic or flank pain symptoms between groups were statistically significant (*P* < .001). Complicating factors were not significantly different between groups. Those with AMS had a urinalysis showing pyuria in 125 patients (83.3%), which was statistically significant compared to those without AMS (n = 344, 76.4%). Urine cultures were obtained more frequently in patients with AMS (86.0%) than in those without AMS (76.7%). A greater proportion of patients with AMS had a history of recurrent UTI (16.7%) versus those without AMS (8.0%) which was a statistically significant difference (*P* = .002). There was no statistically significant difference in DASON empiric antibiotic classification between the AMS and no-AMS groups.

## Discussion

Our results demonstrate that most patients presenting with AMS at University of Kentucky Healthcare are unnecessarily prescribed antibiotic therapy for UTI, as determined by the LMC, and those with AMS are at an even greater risk of receiving antibiotics in the absence of a true indication. These findings are consistent with recent publications reporting overdiagnosis of UTI in AMS^7,8^ and furthers the narrative that prescribing practices for UTI are widely misaligned with peer-reviewed literature. Notably, although only 16.7% of patients with AMS met the LMC, patients without AMS still only met these criteria 33.6% of the time. Although overdiagnosis and treatment is more common in patients with AMS, opportunity for improvement exists in all patient populations. Education efforts for appropriate prescribing in UTI and a clear consensus on how to approach the patient with AMS and suspected UTI are both needed to improve antimicrobial stewardship efforts in medical practice today.

Our study had several limitations. First, the LMC were developed as part of an expert consensus for the treatment of UTI for patients in long-term care facilities. Although the symptoms included in the LMC are similar to those used in standard practice, it could be argued that these criteria have not been validated in the acute-care hospital setting. Second, data regarding the presence of AMS, delirium, and urinary symptoms were solely dependent on provider documentation. Although they are defined differently, “altered mental status” and “delirium” are often used synonymously in practice. Delirium requires the presence of 5 different criteria by the DSM-5 definition, whereas AMS holds a functionally broad definition of any noticeable change in cognitive function from baseline. Because of this gap in commonly used terminology, there exists a greater need for clinical practice to focus identifying true delirium in the setting of UTI. Finally, this study only evaluated adult patients with a urinalysis collected who were actively receiving antibiotics. Because patients not receiving antibiotics were excluded from the study, the extent of antibiotic prescribing could not be evaluated.

This study also had several strengths. This study is the first to compare patients receiving antibiotics for the indication of UTI between those with and those without AMS. Additionally, the LMC used to evaluate this patient population is the only set of published criteria that incorporates the presence of AMS, specifically delirium, as part of a UTI workup. Nevertheless, patients with AMS continued to be prescribed antibiotic therapy in the absence of any combination of qualifying symptoms.

In conclusion, patients with AMS were prescribed antibiotic therapy without any symptoms indicative of UTI at a higher rate than those without AMS, according to the LMC. The overall percentage of patients meeting LMC was impressively low, demonstrating widespread overdiagnosis and overuse of antibiotics in patients with a suspected UTI. Further antimicrobial stewardship efforts should focus on prescriber education and development of clearly defined criteria for patients with and patients without AMS.
